# Investigation of the Role of Anxiety and Depression on the Formation of Phantom Vibration and Ringing Syndrome Caused by Working Stress during Medical Internship

**DOI:** 10.3390/ijerph17207480

**Published:** 2020-10-14

**Authors:** Yu-Hsuan Lin, Kuan-I Lin, Yuan-Chien Pan, Sheng-Hsuan Lin

**Affiliations:** 1 Institute of Population Health Sciences, National Health Research Institutes, Miaoli 35053, Taiwan; yuhsuanlin@nhri.edu.tw (Y.-H.L.); yuanchienpan@gmail.com (Y.-C.P.); 2 Institute of Statistics, National Chiao Tung University, Hsinchu 30010, Taiwan; a9930034759@gmail.com

**Keywords:** phantom vibration syndrome, phantom ringing, hallucination, anxiety, depression, stress, mechanism, mediation analysis

## Abstract

Phantom vibration syndrome (PVS) and phantom ringing syndrome (PRS) are prevalent hallucinations during medical internship. Depression and anxiety are probably understudied risk factors of PVS and PRS. The aim was to evaluate the role of anxiety and depression on the relationship between working stress during medical internship and PVS and PRS. A prospective longitudinal study, consisted of 74 medical interns, was carried out. The severity of phantom vibrations and ringing, as well as anxiety and depression as measured before, at the third, sixth, and 12th month during internship, and two weeks after internship. We conducted a causal mediation analysis to quantify the role of depression and in the mechanism of working stress during medical internship inducing PVS and PRS. The results showed that depression explained 21.9% and 8.4% for stress-induced PRS and PVS, respectively. In addition, anxiety explained 15.0% and 7.8% for stress-induced PRS and PVS, respectively. Our findings showed both depression and anxiety can explain a portion of stress-induced PVS and PRS during medical internship and might be more important in clinical practice and benefit to prevention of work-related burnout.

## 1. Introduction

Phantom vibration syndrome (PVS) and phantom ringing syndrome (PRS) of a mobile phone are two prevalent hallucinations among health care professionals. According to previous studies, 50% to 95% of medical staffs had experienced phantom vibration or phantom ringing [1,2,3,4]. In addition, 4% to 28% medical interns reported severe phantom vibration and 3% to 29% reported severe phantom ringing [1]. Working stress during medical internship is the most well established risk factor for PVS and PRS. The prevalence of phantom ringing among medical interns increased from 27.4% to 87.7% during the one-year internship training, and returned to 54.2% after two weeks when the internship ended. Similarly, the prevalence of phantom vibration among medical interns increased from 78.1% to 95.9% during internship [1]. A similar result was also found in high-tech employees who had experience client related burnout [5]. However, the mechanism by which stress-inducing PVS and PRS arise is still unclear.

Depression and anxiety are probably other understudied risk factors of PVS and PRS. Researchers have found that severe PVS and PRS are also related with high depression and anxiety scores [2], and some researchers have even conceived phantom vibration as belonging to a larger pattern of technology-related anxiety [6]. Several studies have demonstrated working stress, anxiety and depression were associated with PVS and PRS or other hallucination experiences [1,2]. It is plausible that increasing anxiety and depression plays an important role in the mechanism of the stress-induced PVS and PRS development. However, there is still lack of evidence to delineate the causality among depression, anxiety, PVS, and PRS. We herein examined the mediation effects of depression and anxiety in the development of PVS and PRS based on the different timescales and temporal relationship. Phantom vibration and phantom ringing are transient, aberrant perceptions in the absence of a stimulus on a sub-second timescale. By contrast, anxiety and depression are persistent emotional status for days to weeks. In addition, our previous findings supported that the cognitive mechanisms in non-psychotic populations have demonstrated the cognitive underpinnings of auditory hallucination, such as phantom ringing, including abnormal executive inhibition and negative emotions. Therefore, depression and anxiety are more likely to induce PVS and PRS [2].

In this study, we aimed to estimate the role of anxiety and depression on the relationship between working stress during medical internship and PVS and PRS. We conducted causal mediation analysis to quantify the extent to which the incremental severity of PVS and PRS during medical internship can be explained by anxiety and depression.

## 2. Methods

### 2.1. Participants

We recruited 74 volunteers (46 were male) who were medical interns at Chang Gung Memorial Hospital in Taiwan from May 2011 to June 2012. One female participant was lost to follow-up due to non-academic leave. Aged from 23 to 29, all participants were healthy without any mental illnesses. They completed a baseline survey 2 months prior to the beginning their internships and were assessed general demographic factors such as age and gender, Tridimensional Personality Questionnaire (TPQ), Phantom Vibration and Ringing Questionnaire, Beck Depression Inventory (BDI), and Beck Anxiety Inventory (BAI) at the following five stages of medical internship: (1) one week prior to the internship, (2) the 3rd month of the internship, (3) the 6th month of the internship, (4) the 12th month of the internship, and (5) two weeks after their internship. During the medical internship (Stages 2, 3, and 4), each participant worked approximately 86.7 h per week, including 33.5 continuous work hours and 10 nights with on-call per month, but did not work on Stages 1 and 5. All participants provided informed written consent and were awarded by 1000 New Taiwan Dollars (around 33 USD) for fully participation. Since the data has been published in previous papers, the ethical review is not required for the secondary analysis of the existing and de-identified data.

### 2.2. Measurements

#### 2.2.1. Phantom Vibration and Ringing Questionnaire

We assessed the experiences of phantom vibration and phantom ringing with one item, 5-point Likert scales (0–4) respectively. Lin et al. developed these scales [1,2] and we determined any score ≥3 was “positive”, i.e., clinical PVS or PRS, in light of our previous findings [2], which showed that the cutoff point of 2/3 in PVS and PRS could discriminate medical interns with higher and lower anxiety or depression scores. To avoid biasing the respondents, the questionnaire simply stated: ‘‘we are asking you to participate in a research study about cell phones, because in your job you carry one.’’ The questionnaire also asked if the participants had perceived phantom vibration and phantom ringing during the 3th month period. We also asked how bothersome phantom vibration and phantom ringing were during the five stages of internship. In addition, the questionnaire included a severity index of phantom vibration and ringing with five-point Likert items as (0, 1, 2, 3, and 4) with 0 as “having not symptom”, 1 as “having symptom without bothersome”, 2 as “having symptom with a little bothersome”, 3 as “having symptom with bothersome”, and 4 as “having symptom with severe bothersome”.

In this study, we determined the outcome of interest “clinical” or “severe” PVS and PRS. Because of the high prevalence, PVS and PRS might be considered as a “normal” brain mechanism. Furthermore, most (70–95%) medical staff who perceived PVS and PRS reported the perception to be “not at all” or “only a little bothersome” [1,2,3,4]. The “clinical” or “severe” PVS and PRS were PVS and PRS with bothersome or severe bothersome characteristics (Scores = 3 and 4). In contrast, “subclinical” PVS and PRS were no symptom (Scores = 0) or having symptoms without or with a little bothersome characteristics (Scores = 1 and 2).

#### 2.2.2. The Beck Depression and Anxiety Inventories

BDI and BAI are two commonly-used self-administered instruments for detecting symptoms of depression and anxiety. They are both multiple-choice self-report inventory for measuring the severity of depression and anxiety symptoms. BDI is composed of 21 items rated from 0 to 3. In this version, the scores are interpreted as: 0–13 = normal; 14–19 = mild depression; 20–28 = moderate depression; and 29–63 = severe depression. Similarly, BAI is composed of 21 items and anxiety is scored in the following manner: 0–7 = minimal anxiety; 8–15 = mild anxiety; 16–25 = moderate anxiety; and 26–63 = severe anxiety.

### 2.3. Statistical Analysis

**Figure 1:**
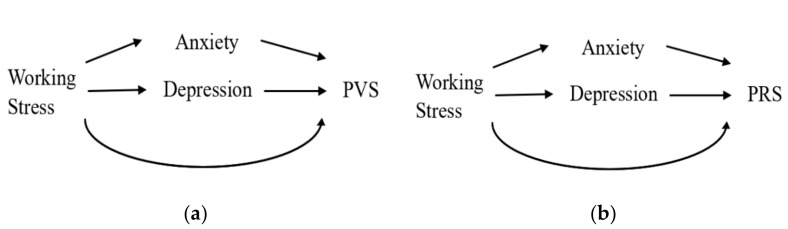
(**a**) Causal relationship among working stress during medical internship, anxiety, depression, and phantom vibration syndrome (PVS); (**b**) Causal relationship among duty stress, anxiety, depression, and phantom ringing syndrome (PRS).

We used a causal mediation analysis to investigate the role of depression and anxiety in the mechanism of working stress during medical internship inducing PVS and PRS. According to previous literatures, it is assumed that PVS, PRS, anxiety and depression are caused by working stress during medical internship [1,2]. It is also assumed that anxiety and depression are the cause of PVS and PRS. The causal relationships among working stress during medical internship, PVS and PRS, and anxiety and depression, as shown in Figure 1. The working stress was examined by the differences of work hours and night shift between Stages 2, 3, 4 (medical internship) and Stages 1, 5 (one week prior to internship, and two weeks after internship). Since depression and anxiety measured by BDI and BAI can be treated as two relative independent variables [7], the pathway mediated by depression and anxiety were assumed to be paralleled. The total effect of the working stress during medical internship on PVS and PRS could be decomposed into three parts: (1) the part involving the change of anxiety score; (2) the part involving the change of depression score; and (3) the part not involving that change of either anxiety or depression score. We can conduct mediation analysis for two times, for each time we treated anxiety or depression as the only one mediator of interest and evaluate the mediated effect and proportion mediated. As a result, mediation analysis was conducted for four times: (1) anxiety as the mediator and PVS as the outcome; (2) depression as the mediator and PVS as the outcome; (3) anxiety as the mediator and PRS as the outcome; and (4) depression as the mediator and PRS as the outcome. For each mediation analysis, we built the following two regression models for an outcome of interest (PVS or PRS) and a mediator of interest (anxiety or depression, respectively):

(1) $$M_{ij}=\;\alpha +a_{i}+\beta A_{ij}+\gamma C_{i}+\epsilon_{i}$$

(2) $$logit\left(P(Y_{ij}=1)\right)=\lambda +d_{i}+\psi A_{j}+\phi M_{ij}+\varsigma C_{i}$$

where individual indicator i = 1, …, 73, time indicator j = 0, 1, 5. $C_{i}$ is the baseline covariates of each individual i, including gender and personality trait. For individual i measured at time j, the exposure (duty status) is denoted as $A_{ij}$, the mediator (anxiety or depression score) as $M_{ij}$, and the outcome (PVS or PRS) as $Y_{ij}$. Generalized linear mixed models with random effect ($a_{i}$ and $d_{i}$) for intercept were applied to adjust the autocorrelation among the observations for the same individual with repeated measurements. All analyses were performed by mediation using the mediation. R package (R Core Team, Vienna, Austria), developed by Tingley et al. [8] on R version 3.3.2.

## 3. Results

**Figure 2:**
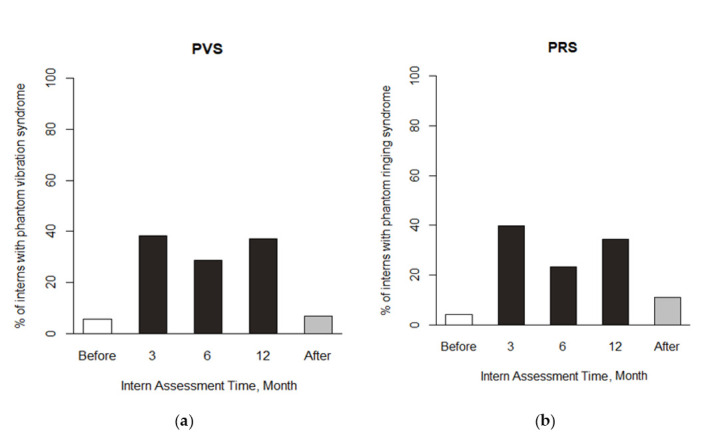
(**a**) The proportion of interns who reported clinical phantom vibration syndrome (PVS) before and after the internship and at the 3rd, 6th, and 12th months during the internship. (**b**) The proportion of interns who reported clinical phantom ringing syndrome (PRS) before and after the internship and at the 3rd, 6th, and 12th months during the internship.

Figure 2 shows the prevalence of clinical PVS and PRS trends on five stages of internship. The prevalence of PVS under the third, sixth, and 12th internship stages was 38.4%, 28.8%, and 37% respectively. Similarity, PRS under the third, sixth, and 12th internship stages was 39.7%, 23.3%, and 34.2%, respectively. In contrast, the PVS prevalence before and after internship was 5.5% and 6.8%, respectively, while the PRS prevalence before and after internship was 4.1% and 11%, respectively, which was obviously different from those within internship. Therefore, we re-categorized the third, sixth, and 12th internship into the same group for the following analysis to increase the statistical power. The crude associations among duty status, anxiety, depression, PVS, and PRS are shown in Table A1 in Appendix A. The correlation is among 21.5% to 55.7% (*p*-values are all less than 0.001).

The result of the causal mediation analysis for the duty’s effect on PVS mediated by anxiety and depression is shown in Table 1. Working stress during medical internship increased PVS for 0.287 (95% CI = 0.189–0.378) and 0.291 (95% CI = 0.195–0.382). The two estimates were almost identical but slightly different because our methods were the numerical solution based on Monte-Carlo simulation [8]. By treating anxiety score as the mediator, the total effect was further decomposed into the effect mediated by the change of anxiety score as 0.036 (95% CI = 0.010–0.067) (mediation effect) and the effect not mediated through anxiety as 0.252 (95% CI = 0.154–0.339) (alternative effect). By treating depression score as the mediator, the total effect was further decomposed into the effect mediated by the change of depression score as 0.065 (95% CI = 0.016–0.116) (mediation effect) and the effect not mediated through depression as 0.226 (95% CI = 0.123–0.322) (alternative effect). The change in anxiety explained the total effect of working stress on an increase of PVS by around 7.8 % (95% CI= 2.1–18.9%), while depression explained the total effect by around 15.0 % (95% CI= 3.8–31.9%).

**Figure 3:**
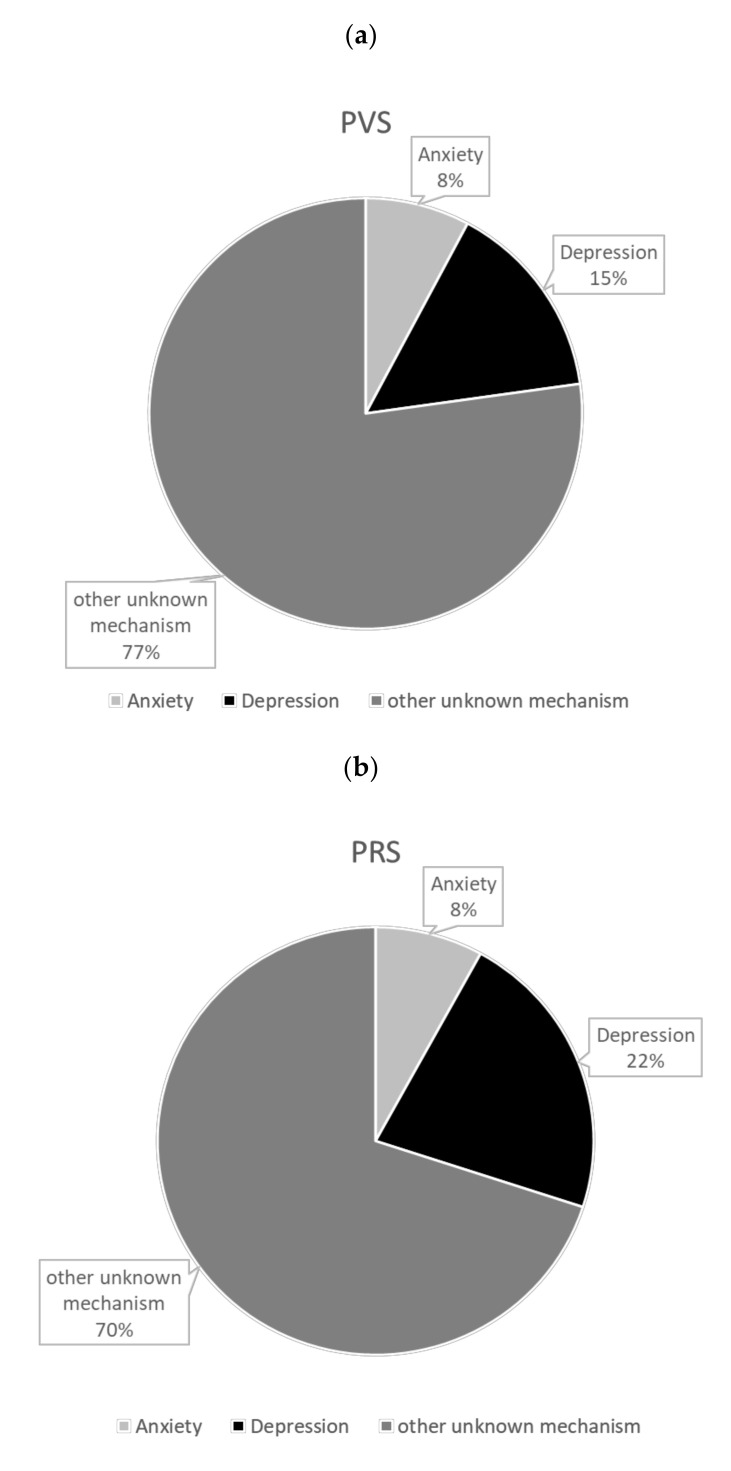
(**a**) Proportion of stress-induced phantom vibration syndrome (PVS) explained by the change of depression and anxiety score. (**b**) Proportion of stress-induced phantom ringing syndrome (PRS) explained by the change of depression and anxiety score.

The result of the causal mediation analysis for the effect of working stress on PRS mediated by anxiety and depression is shown in Table 2. The total effect of working stress during medical internship on PRS was around 0.282 (95% CI = 0.192–0.376). The path through anxiety explains the overall effect of working stress during medical internship on PRS for around 8.4% (95% CI = 2.8–19.0%), while the path through depression explains the overall effect of working stress during medical internship on PRS for around 21.9 (95% CI = 11.4–41.6%). The result is also visualized in Figure 3.

## 4. Discussion

This study is the first to identify and quantify the role of depression and anxiety in mechanism of stress-induced PVS and PRS. We showed that depression explained 21.9% and 8.4% for stress-induced PRS and PVS, respectively; while anxiety explained 15.0% and 7.8% for stress-induced PRS and PVS, respectively. Although PVS and PRS used to be considered as anxiety-related symptoms instead of depression [6], or even “ringxiety” for PRS [9], our findings emphasized the importance of depression by showing that higher proportion mediated by depression than anxiety for both PVS and PRS. The prevalence of clinical depression was high among resident physicians (28.8%) [10] and medical students (14.3%) [11]. It was also reported that physicians’ depression was highly associated to poor-quality patient care and increased medical errors [12]. Therefore, our study showed the potential causal relationship among working stress, depression, PVS and PRS, proposing a future direction that PVS and PRS might also be important indicators to health care professionals’ mental health.

The mediation effects of depression and anxiety in the development of PVS and PRS provided a new evidence to clarify the pathways of the hallucinations formation from stressors mediated by emotional regulation. Although PVS and PRS were not typical hallucinations in patients with psychotic disorders, these findings are still useful to understand the psychopathology induced by working stress in our daily life. One of the possible mechanisms are depression symptoms significantly correlated to sensory sensitivity in patients with affective disorders [13]. In addition, PVS and PRS might be associated with interns’ hypervigilance, which is a typical anxiety symptom in stress-related disorders such as post-traumatic stress disorder.

The theory of dopamine system dysregulation has been nearly universally seen as having a central role in the development of psychotic symptoms such as hallucinations [14,15,16]. The dysregulated dopamine system fires and releases dopamine independent of cue and context [17]. This event-linked, self-terminating action occurs on a sub-second timescale, and has already been shown by measuring the burst firing of dopamine neurons and the release of phasic dopamine in limbic regions [18,19]. The dysregulated dopamine system in hallucinations on a sub-second timescale might correspond to the experience of phantom vibrations and ringing, as medical interns heard the transient, vague ringing but promptly recognized their misperception after checking their mobile phones. This mechanism was enhanced during medical internship since emotionally salient stimuli, such as depression and anxiety, were experienced. The lowered threshold for hallucinating has been observed in individuals with bereavement and a hypervigilant state [20]. The present findings provided a new evidence to clarify the overlapping circuits that mediate stress responses, emotional learning and reward processing [21].

In this study, we not only identified interns’ experience of phantom vibration and phantom ringing, but also assessed the severity with bothersome or very bothersome. We found that the incidence of severe PVS and PRS (also termed as clinical PVS and PRS) were similar at each time point. Specifically, 26.8% to 38.4% of the interns experienced clinical phantom vibration and 23.3% to 39.7% experienced clinical phantom ringing during internship. Only 5.5% to 6.8% and 4.1% to 11% of the interns experienced clinical phantom vibration and phantom ringing respectively before and after internship. These trends differed from our previous studies, showing that medical interns experienced more PVS than PRS in the same sample without this severity assessment. PVS and PRS were prevalent hallucinations during medical internship, and they might be considered as a “normal” brain mechanism to working stress. In addition, there was no significant difference between medical interns who experienced PVS and PRS or not without the severity assessment. Our results in the present study supported the theory that in addition to surveying the experience of phantom vibration and ringing, the severity assessment is necessary to link this phenomenon to clinical implication. Furthermore, the recovered proportion of interns experienced clinical PVS and PRS two weeks after the internship (6.8% and 11%) close to the baseline proportion (5.5% and 4.1%) before internship, suggested that medical interns should have at least two weeks of annual leave to mitigate anxiety and depression symptoms from working stress during the internship to the extent of baseline [22].

Mechanism investigation in this study was analyzed by causal mediation analysis. Compared with structural equation modeling, causal mediation analysis can be applied to non-linear outcome and mixed effect model, which is fitting our study design. In addition, causal mediation analysis also provides causal interpretation for both total effect and mediated effect, given all assumptions are not violated. Before making further inference, several limitations for methodology should be noticed. First, the confounding between anxiety/depression and PVS/PRS was assumed to be eliminated after controlling gender and personality. More potential confounders such as individual stressful life event should be collected in future studies. Secondly, the paths mediated by anxiety and depression were assumed to be independent. Although previous studies showed the anxiety and depression measured by BAI and BDI look orthogonal to each other, a more generalized mediation analysis should be developed and applied to estimate the path specific effect under a more complex causal relation. Finally, anxiety and depression were assumed to be the cause of PVS and PRS, but they were measured at the same time. Although BAI and BDI represented long-term average statuses of anxiety and depression while the Phantom Vibration and Ringing Questionnaire represented a short term recall of PVS and PRS episodes, it will be much better to measure anxiety and depression prior to measuring PVS and PRS, in order to ensure the temporality.

In addition to the limitations in methodology, several limitations in substantive part are also noteworthy. First, a large portion of the mechanism still cannot be explained by anxiety and depression. One possible mechanism might be Hypothalamus-Pituitary-Adrenal (HPA) induced by stress, which further stimulate the development of PVS and PRS. More factors representing other possible pathways should be collected for comprehensive understanding for mechanism in further study. Secondly, all measurements were reported by participants themselves, which might be subject to recall bias when reporting PVS and PRS during each three-month course. Our study also lacks a more structured interview to confirm PVS, PRS, depression, and anxiety. A more objective method is necessary for mechanism investigation. For example, the well-validated neurophysiological measurements of autonomic modulation, such as heart rate variability [23,24,25,26], may also make it possible to evaluate the role of HPA axis in the formation of these hallucinations. Moreover, our study participants were all medical interns, which limited the generalizability of our findings. As a trade-off, the internal validation of our result is relatively solid due to the homogeneity of this study population and the intervention. For example, all medical interns had been exposed to the same training course and identical working stress. In addition, there was only one loss-of-follow-up in this cohort. Finally, we estimated work hours through the official schedule of the hospital but did not measure the actual work hours of each participant. Quantifying the stressors during the internship, such as using the mobile app to automatically record medical interns’ work hours real-time [26,27], could strengthen our understanding of the relationship between stress and these hallucinations.

## 5. Conclusions

Our findings showed both depression and anxiety can explain a portion of stress-induced PVS and PRS during medical internship. Our study also emphasized the importance of depression by showing that a higher proportion was mediated by depression than anxiety for both PVS and PRS. A dimensional approach to identify the phantom vibrations and ringing associated with anxiety and depression during medical internship might be more appropriate in clinical practice and of more benefit to prevention of work-related burnout.

## Figures and Tables

**Table 1:** Causal mediation analysis for the mechanism of the duty’s effect on PVS mediated by anxiety and depression.

Mediator of Interest	Anxiety				Depression			
Effect	Estimate	95% CI		*p* Value	Estimate	95% CI		*p* Value
Total effect	0.287	0.189	0.378	<0.001	0.291	0.195	0.382	<0.001
Mediation effect	0.036	0.010	0.067	0.008	0.065	0.016	0.116	0.006
Alternative effect	0.252	0.154	0.339	<0.001	0.226	0.123	0.322	<0.001
Proportion mediated	0.078	0.021	0.189	0.008	0.150	0.038	0.319	0.006

**Table 2:** Causal mediation analysis for the mechanism of the duty’s effect on PRS mediated by anxiety and depression.

Mediator of Interest	Anxiety				Depression			
Effect	Estimate	95% CI		*p* Value	Estimate	95% CI		*p* Value
Total effect	0.282	0.192	0.376	<0.001	0.280	0.191	0.365	<0.001
Mediation effect	0.039	0.013	0.072	0.002	0.094	0.052	0.142	<0.001
Alternative effect	0.243	0.156	0.331	<0.001	0.186	0.098	0.269	0.002
Proportion mediated	0.084	0.028	0.190	0.002	0.219	0.114	0.416	<0.001

## Appendix A

**Table A1:** Cohan Kappa value among working stress, anxiety, depression, phantom vibration syndrome, and phantom ringing syndrome.

		1	2	3	4	5
1	Working stress	-	0.355 (0.257–0.453)	0.262 (0.164–0.361)	0.248 (0.156–0.339)	0.215 (0.124–0.307)
2	Anxiety		-	0.513 (0.425–0.601)	0.171 (0.076–0.265)	0.228 (0.135–0.321)
3	Depression			-	0.167 (0.065–0.268)	0.205 (0.104–0.306)
4	Phantom vibration syndrome				-	0.557 (0.452–0.663)
5	Phantom ringing syndrome					-
